# Protocol of the optimal study: Optimization of polypharmacy in geriatric oncology - A randomized controlled trial

**DOI:** 10.1186/s12885-023-10812-7

**Published:** 2023-04-18

**Authors:** Ben Schöttker, Li-Ju Chen, Reiner Caspari, Hermann Brenner

**Affiliations:** 1 Division of Clinical Epidemiology and Aging Research, German Cancer Research Center (DKFZ), Im Neuenheimer Feld 581, 69120 Heidelberg, Germany; 2 Rehabilitation Clinic Niederrhein, Hochstraße 13-19, 53474 Bad Neuenahr-Ahrweiler, Germany; 3 Division of Preventive Oncology, German Cancer Research Center (DKFZ) and National Center for Tumor Diseases (NCT), Im Neuenheimer Feld 460, 69120 Heidelberg, Germany; 4 German Cancer Consortium (DKTK), German Cancer Research Center (DKFZ), Im Neuenheimer Feld 280, 69120 Heidelberg, Germany

**Keywords:** Randomized controlled trial, Polypharmacy, Potentially inappropriate medication, Cancer, Rehabilitation, Quality of life

## Abstract

**Background:**

Polypharmacy is very common in older cancer patients and these patients are particularly vulnerable to drug-drug interactions and adverse drug reactions because they often receive chemotherapy and symptom-relieving agents.

**Methods:**

The primary aim of the randomized, controlled *Optimization of Polypharmacy in Geriatric Oncology (OPTIMAL)* trial is to test whether an advisory letter with the results of a comprehensive medication review conducted with the Fit fOR The Aged (FORTA) list to the caring physician in rehabilitation clinics improves the quality of life (QoL) of older cancer patients exposed to polypharmacy more than usual care. The FORTA list detects medication overuse, underuse, and potentially inappropriate drug use among older adults. In the oncology departments of approximately 10 German rehabilitation clinics, we aim to recruit 514 cancer patients (22 common cancers; diagnosis or recurrence requiring treatment in the last 5 years; all stages) who are ≥ 65 years old, regularly take ≥ 5 drugs, and have ≥ 1 medication-related problem. All necessary information about the patients will be provided to a pharmacist at the coordinating center (German Cancer Research Center, Heidelberg), who will perform randomization (1:1) and conduct the medication review with the FORTA list. For the intervention group only, the results are sent by letter to the treating physician in the rehabilitation clinics, who shall discuss medication changes with the patient at the discharge visit, as well as implement them afterwards and disclose them in the discharge letter to the general practitioner. The control group gets the usual care provided in German rehabilitation clinics, which usually does not include a comprehensive medication review but can include medication changes. Patients will be blinded, as they cannot know whether proposed medication changes were part of the study or part of usual care. Study physicians cannot be blinded. The primary endpoint will be the EORTC-QLQ-C30 global health status/QoL score, assessed via self-administered questionnaires 8 months after baseline.

**Discussion:**

If the planned study shows that a medication review with the FORTA list improves the QoL of older cancer patients in oncological rehabilitation more than usual care, it would provide the necessary evidence to translate the trial’s findings into routine care.

**Trial registration:**

German Clinical Trials Register (DRKS): DRKS00031024.

## Background

The number of older cancer patients in Germany is relatively large and it is expected to increase further due to demographic changes and trends towards improved cancer survival rates by adopting the best practice in cancer treatment and management [1]. In Germany, the majority of cancer patients receives at least 3 weeks of in-patient rehabilitation shortly after primary cancer therapy to support them in coping with functional impairments that may occur as a result of the cancer therapy [2].

The prevalence of multimorbidity and polypharmacy strongly increases with age, making treatment of older cancer patients complex and challenging. For example, although guidelines exist for the management of many major chronic diseases, adherence to these guidelines may lead to polypharmacy and adverse drug-drug and drug-disease interactions in the presence of multiple chronic conditions [3]. Older cancer patients are particularly prone to the unintended consequences of polypharmacy because they often receive chemotherapy and symptom-relieving agents, which may entail additional risk for drug-drug-interactions and adverse drug reactions [4].

The term “polypharmacy” is mostly defined by 5 or more concurrently prescribed drugs [5]. For example, every second colorectal cancer patient aged 65 years or older was exposed to polypharmacy in a German study, and polypharmacy was significantly inversely associated with overall and cancer-specific survival although potential confounding was well controlled by adjustment for all other major risk factors for poor cancer survival [6].

With an increasing number of prescribed drugs in general, the likelihood for potentially inappropriate prescribing (PIP) increases. PIP is an umbrella term for medication overuse, underuse, and the use of potentially inappropriate medication (PIM) in older adults. An assessment tool for all these three aspects of PIP is the Fit fOR The Aged (FORTA) list [7]. A German study applied the FORTA list and showed how common these medication problems are among older colorectal cancer patients aged 65 years and older [8]. The prevalence of PIM, medication over-, and underuse was 52.6%, 66.7% and 48.7%, respectively.

### Objectives

The aim of the OPTIMAL study is to test with a single-blinded, randomized, controlled trial (RCT) design, whether an advisory letter with the results of a comprehensive medication review conducted with the FORTA list to the treating physician in rehabilitation clinics improves the global quality of life (QoL) of older cancer patients exposed to polypharmacy more than usual care. Secondary endpoints will be QoL sub-domains, hospitalizations, falls, mortality, fatigue, frailty, and medication quality.

A further aim of the OPTIMAL study is to establish a biobank with blood sample based data (including biomarker and genetic data) and other data collected in this study to investigate risk and protective factors for the development and unfavorable course of cancer. The term “unfavorable course” is broadly defined and includes death, hospitalizations, concomitant diseases, symptoms and QoL, and difficulties in performing activities of daily living.

## Methods/Design

The trial protocol has been developed in line with the SPIRIT guideline (Standard Protocol Items: Recommendations for Interventional Trials) [9]. This manuscript is based on the trial protocol version 4.0 from April 7, 2023. At the time of submission of the trial protocol, recruitment for the OPTIMAL study is planned to start in June 2023 and to be completed within 3 years.

### Study design

**Fig. 1:**
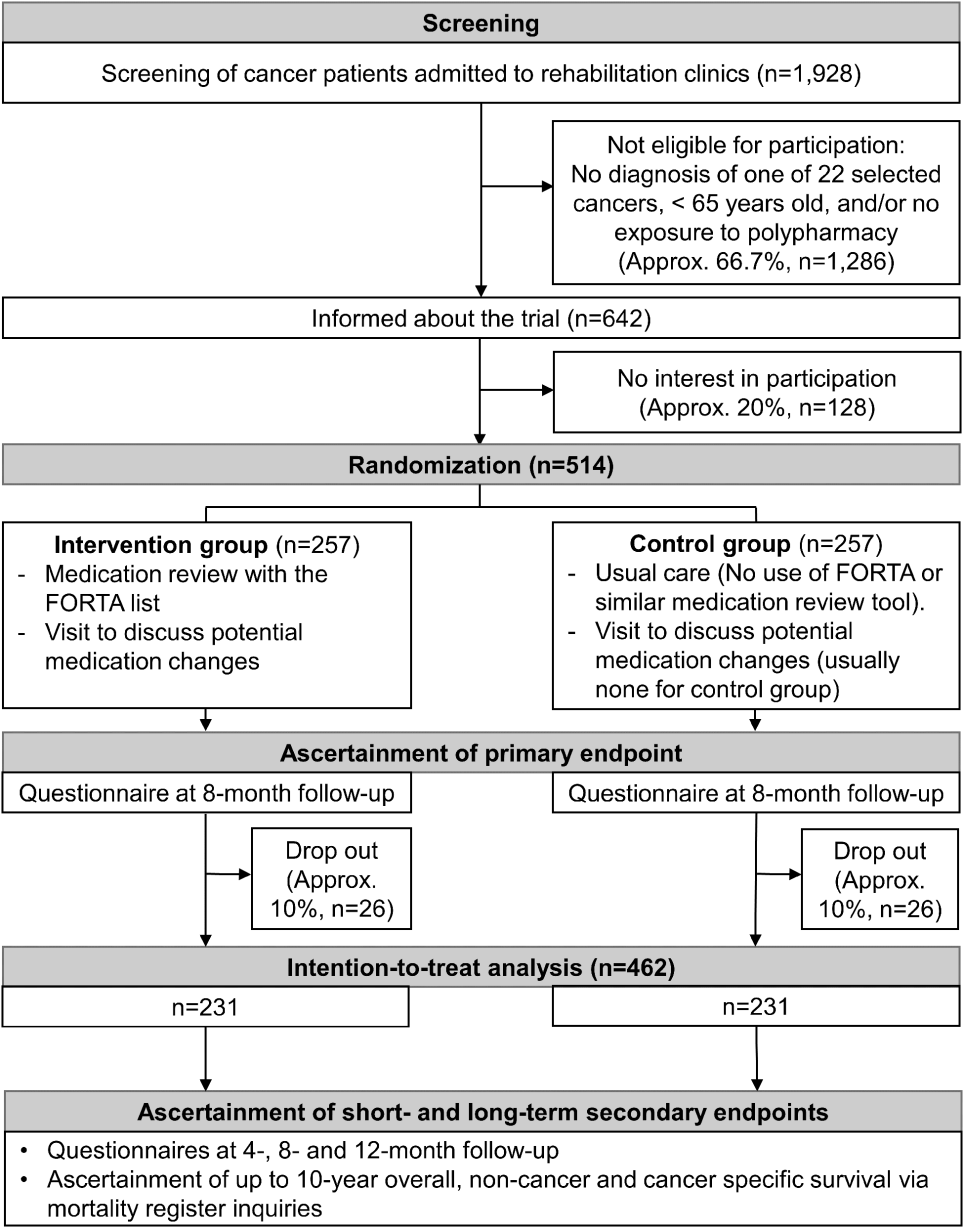
Flow-chart of the OPTIMAL study. Abbreviations: FORTA, Fit fOR The Aged

The OPTIMAL study is a national, multicenter, prospective, single-blinded, randomized, controlled trial aiming to test the superiority of an intervention over usual care. Figure 1 shows the schematic flow-chart of the study. By applying a parallel group design, the participants are randomly assigned in a 1:1 ratio either to the intervention group or to the usual care (control) group.

Recruitment is planned in approximately 10 rehabilitation clinics located all over Germany. The coordinating center is the Division of Clinical Epidemiology and Ageing Research, German Cancer Research Center, Heidelberg, which will not participate in the recruitment of study participants but will conduct follow-ups after the rehabilitation clinic phase of the study.

### Endpoints

The primary endpoint of the study is the change of the European Organisation for Research and Treatment of Cancer’s Quality of Life Questionnaire’s Core 30 items (EORTC’s QLQ-C30 [10]) global health status (GHS)/QoL score. The GHS/QoL score can change in the time between filling of the baseline questionnaire (which takes place in the first 1–2 weeks of the rehabilitation clinic stay) and filling of the 8-month follow-up questionnaire (which takes place in study month 8–9). The GHS/QoL score is based on the following two questions: “How would you rate your overall health during the past week?” and “How would you rate your overall QoL during the past week?”, both of which have a scale from 1 (“very poor”) to 7 (“excellent”). According to the EORTC-QLQ-C30 manual, the two numbers are being summed up, the sum is being divided by two and subsequently standardized to a scale from 0 to 100 points.

The following Table 1 lists all endpoints that will be examined in the OPTIMAL study and the time-points of their assessment. Secondary endpoints will be the change of medication quality (total FORTA score and its sub-scores [7]) from rehabilitation clinic admission to discharge in week 3, other time-points of assessments of the GHS/QoL score (at 4- and 12-month follow-ups, which take place in study months 4–5 and 12–13, respectively), the other domains of the EORTC-QLQ-C30 including physical and cognitive functioning as well as symptom scores that often reflect adverse drug reactions (nausea, constipation diarrhea), dizziness, fatigue, frailty, falls, hospitalizations, as well as overall, non-cancer, and cancer-specific survival [10–12].

**Table 1:** Outcomes and their Operationalization

Outcomes	Ascertainment/Operationalization	Time of assessment				
		Week 3	Month 4–5	Month 8–9	Month 12–13	Year 1–10
Total medication quality	Total FORTA score	x		x		
Medication underuse	FORTA medication underuse sub-score	x		x		
Medication overuse	FORTA medication overuse sub-score	x		x		
PIM use	FORTA PIM use sub-score	x		x		
Global health status	EORTC-QLQ-C30 questions: 29 + 30		x	x (primary)	x	
Physical functioning	EORTC-QLQ-C30 questions: 1–5		x	x	x	
Role functioning	EORTC-QLQ-C30 questions: 6,7		x	x	x	
Emotional functioning	EORTC-QLQ-C30 questions: 21–24		x	x	x	
Cognitive functioning	EORTC-QLQ-C30 questions: 20,25		x	x	x	
Social functioning	EORTC-QLQ-C30 questions: 26,27		x	x	x	
Dyspnoea	EORTC-QLQ-C30 question: 8		x	x	x	
Pain	EORTC-QLQ-C30 questions: 9, 19		x	x	x	
Insomnia	EORTC-QLQ-C30 question: 11		x	x	x	
Appetite loss	EORTC-QLQ-C30 question: 13		x	x	x	
Nausea and vomiting	EORTC-QLQ-C30 questions: 14,15		x	x	x	
Constipation	EORTC-QLQ-C30 question: 16		x	x	x	
Diarrhoea	EORTC-QLQ-C30 question: 17		x	x	x	
Dizziness	Self-developed					
Fatigue	EORTC-QLQ-C30 questions: 10,12,18		x	x	x	
Physical fatigue	EORTC-FA12 questions: 1–5		x	x	x	
Emotional fatigue	EORTC-FA12 questions: 6–8		x	x	x	
Cognitive fatigue	EORTC-FA12 questions: 9–10		x	x	x	
Interference of fatigue with daily life	EORTC-FA12 question: 11		x	x	x	
Frailty	FRAIL-SCALE				x	
Falls	Frequency, self-reported				x	
Hospitalizations	Frequency, self-reported				x	
All-cause mortality	All deaths					x
Cancer mortality	ICD-10 codes C00-C97					x
Non-cancer mortality	All ICD-10 codes except C00-C97					x

Abbreviations: European Organisation for Research and Treatment of Cancer’s Quality of Life Questionnaire’s Core 30 items; EORTC-QLQ-FA12, EORTC’s Quality of Life Module Measuring Cancer Related Fatigue with 12 items; FORTA, Fit fOR The Aged; FRAIL-SCALE, Fatigue, Resistance, Ambulation, Illnesses, & Loss of Weight; ICD, International Classification of Diseases; PIM, potentially inappropriate medication

### Study population

The target population is older cancer patients (≥ 65 years), exposed to polypharmacy, who undergo in-patient rehabilitation in a cooperating clinic for at least 3 weeks. The target population will be representative for older, multi-morbid cancer patients in the German rehabilitation setting because it will be carried out as a multi-center study and the in- and exclusion criteria are restricted to the necessary minimum (e.g., by excluding only rare cancer sites, and by not excluding specific cancer stages or patients with specific cancer treatments).

In detail, the following inclusion criteria will be applied in the OPTIMAL study:

1. Diagnosis or recurrence requiring treatment of one of 22 selected, frequent cancers (International Classification of Diseases (ICD)-10 codes) in the last 5 years: esophagus (C15), stomach (C16), small intestine (C17), colorectal (C18-20), anus (C21), liver (C22), gallbladder/other biliary tract (C23-24), pancreas (C25), lung (C33-C34), malignant melanoma (C43), breast (C50), vulva (C51), cervix (C53), uterus (C54-55), ovary (C56), prostate (C61), kidney (C64), bladder (C67), Hodgkin lymphoma (C81), non-Hodgkin lymphoma (C82-C88), multiple myeloma (C90), and leukemia (C91-C95).
2. Age ≥ 65 years.
3. Exposure to polypharmacy, defined as ≥ 5 chronically, concurrently used active substances (including on-demand medication if used at least once per month). Food supplements (except vitamin D), homeopathic drugs, anthroposophical drugs, herbal drugs without systemic action and other non-systemic acting drugs (for local action in the skin, eyes, ears, nose or throat, only) are not counted because they do not cause (systemic) adverse drug reactions if they are not overdosed. This definition of “clinically relevant polypharmacy” has been published previously [13].
4. At least 3 weeks of in-patient rehabilitation in a cooperating clinic are planned.
5. Sufficient capabilities for informed consent.

The following exclusion criteria will be applied in the OPTIMAL study:

1. No written informed consent to participate.
2. No sufficient mental and physical capabilities to fill self-administered questionnaires.
3. No sufficient knowledge of the German language.
4. Missing information needed to conduct the medication review with the FORTA list (medical diagnoses and drugs used at the start of rehabilitation).
5. No medication related problem at baseline (FORTA score = 0).
6. Missing baseline information needed for the primary endpoint (change in EORTC-QLQ-C30 GHS/QoL scale).

### Measures during the course of the study

**Fig. 2:**
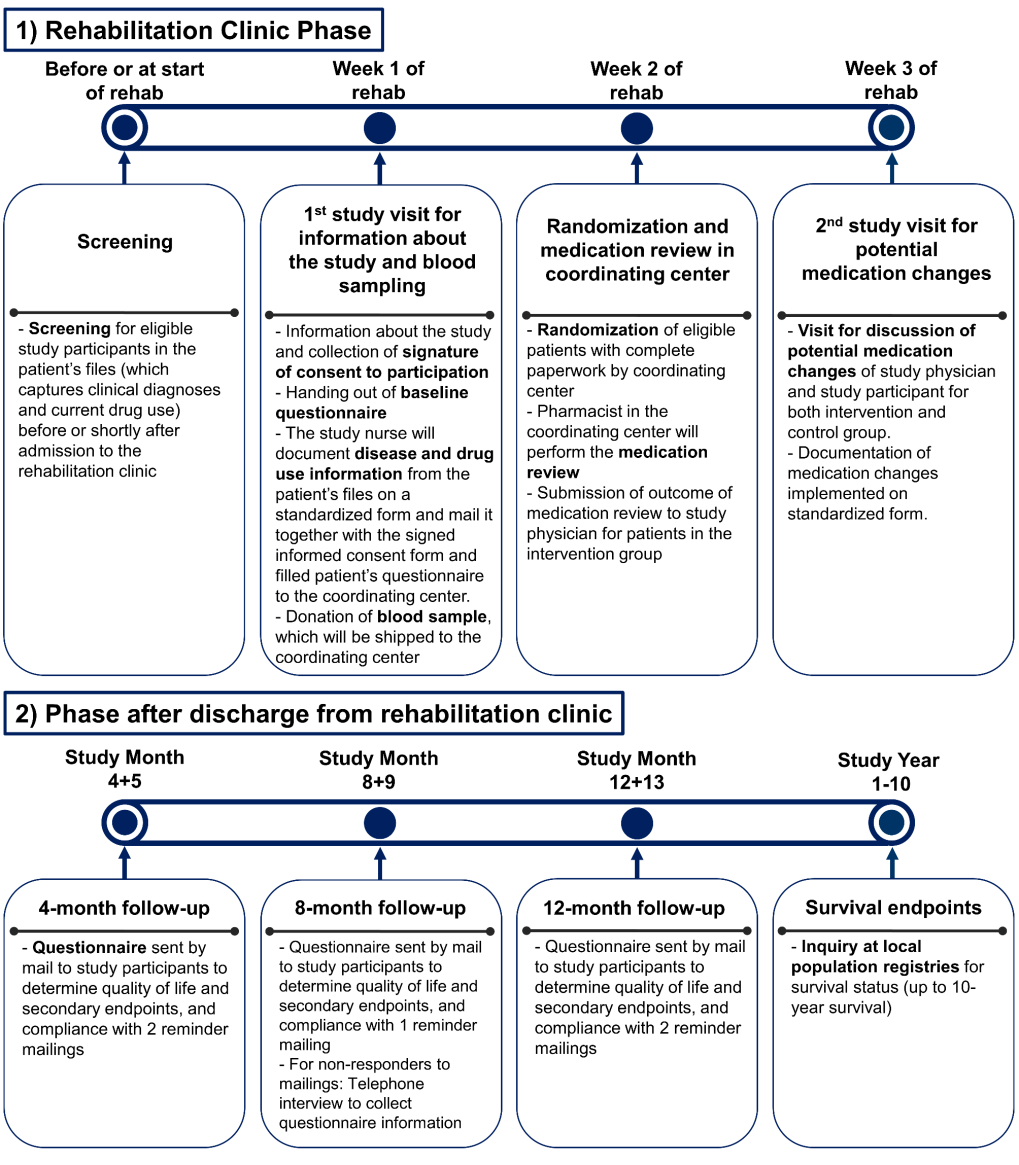
Contacts with study participants during the course of the study and their scope

The contacts with study participants during the course of the study and their scope is shown in Fig. 2.

#### Screening (before or at the start of rehabilitation)

Screening for eligible study participants will be done by a study nurse or a study physician via checking the patient’s files (which captures clinical diagnoses and current drug use) before or shortly after their admission to the rehabilitation clinic with a standardized screening checklist stating the in- and exclusion criteria.

#### 1st study visit for information about the study (week 1 of rehabilitation)

During the first appointment in the rehabilitation clinic, eligible patients will be informed about the OPTIMAL study by a study nurse or a study physician and will receive the printed study information and baseline questionnaire. They will have the opportunity to ask questions and will be given enough time to consider their participation. A written informed consent to participate in the OPTIMAL study will be obtained.

Study participants will be asked to fill the baseline questionnaire in the next 2 days. The questionnaires are used for the collection of important parameters (e.g. drug use, medical diagnoses, vaccinations, lifestyle factors, baseline levels of endpoint scales (e.g. EORTC-QLQ-C30), and information needed to be able to contact the study participants for the follow-ups (name, sex, birthday, address and phone number). A study physician/nurse should ensure that the study participant completely fill the questionnaire. Furthermore, the study nurse/physician will copy information about medical diagnoses and prescribed drugs from the patient’s files to the appendix of the standardized screening checklist. Towards the end of rehabilitation week 1, the study nurse/physician will mail the screening checklist, the signed informed consent form, and the patient’s questionnaire to the coordinating center.

#### Blood sampling (week 1 of rehabilitation)

Usually in the morning after the first visit, 38.6 ml blood will be collected in 6 tubes (1 × 2.6 ml EDTA plasma, 1 × 9 ml EDTA plasma, and 3 × 9 ml serum) from each patient. All tubes will be mailed to the laboratory of the coordinating center with DHL express while ensuring a temperature between 2 and 8 °C. The blood samples will be stored in the coordinating center in freezers at -80 °C to serve as a biobank for future research.

#### Randomization, blinding, and medication review by pharmacist (week 2 of rehabilitation)

The pharmacist in the coordinating center will check correct inclusion into the study and randomize the study participants 1:1 to the intervention and control group. Computer-generated randomization lists will be prepared by the pharmacist before study initiation with randomizer.at, which will be stratified by recruiting center using permuted blocks of random sizes. The block sizes will not be disclosed, to ensure concealment. The allocation sequence will be stored in a locker whose keys will only be available for the pharmacist and his/her deputy. The randomization numbers will be linked to the study participant’s IDs. The study participant’s IDs are used to label the informed consent forms, questionnaires, and all other study materials for a specific patient. The study materials will be packed for each study participant’s ID in the coordinating center and shipped to the rehabilitation clinics. Study participants will get their ID with the package containing the study materials after giving their written informed consent to participate in the study. Staff in the recruiting study centers and the study participants will not see the randomization lists or any randomization number at any time. Unblinding of study participants will be prohibited until end of study month 13 (end of time for collection of 12-month follow-up questionnaires).

The pharmacist in the coordinating center will further perform the medication review with the 4^th^ version of the FORTA list updated in 2021 [7]. Medical diagnoses and drug use obtained from two sources (patient’s medical files and patient’s questionnaires) will be combined to ensure a high completeness.

The use of the FORTA list will be done in two steps:

1st step: Each of the medical diagnoses will need to be checked. The FORTA list comprises the 30 most frequent medical diagnoses and a list of frequently used active substances for each diagnosis. The active substances have a rating from A (indispensable) to D (avoid). The pharmacist will then compare the present medication with the rated active substances in the FORTA list and rates each drug/diagnosis found as follows:

- Appropriate use: The medication is indicated; a drug classified as A (indispensable) or B (beneficial) is prescribed.
- PIM use: The medication is indicated but a drug classified as C (questionable) or D (avoid) in the FORTA list is given despite available classes A (indispensable) or B (beneficial) alternatives.
- Underuse: An indication is not treated.

2nd step: Drugs not rated in the first step will then be checked and rated as follows:

- Overuse: The medication is prescribed in the absence of an appropriate indication.
- Appropriate use: The medication is appropriately prescribed according to clinical guidelines for a disease not listed in the FORTA list, which is limited to 30 diseases.

The pharmacist will subsequently calculate the FORTA sub-scores for underuse, overuse, and PIM use for every patient and sum the three scores up to obtain the total FORTA score. Each medication underuse and overuse scores 1 point. Each PIM use scores 2 points.

For patients randomized to the intervention group, the pharmacist will fax an advisory letter to the study physicians stating suggested medication changes to overcome PIM use (by replacing drugs with a C or D rating by drugs with an A or B rating), underuse (by adding drugs with an A or B rating), and overuse (by withdrawal of drugs without indications). If the FORTA list suggests no medication changes, the letter will contain only the sentence: “No medication changes suggested”. For patients randomized to the control group, the letter will include the same sentence: “No medication changes suggested”.

Thus, the study physicians will be partly blinded because they cannot know the randomization group for patients for whom they received the sentence “No medication changes suggested” in the advisory letter from the coordinating center. However, they could still correctly guess that patients with medication changes suggested in the letter are in the intervention group. Thus, due to the nature of the intervention, the study physicians cannot be fully blinded and the study design is only a single-blinded study (only study participants will be blinded).

#### 2nd study visit for potential medication changes (week 3 of rehabilitation)

The study physician will have a visit with the study participants to discuss his/her medication. This should take place for both the intervention and control group to avoid accidental unblinding of study participants. For study participants in the intervention group, the study physician can follow the advice from the letter of the pharmacist, who conducted the comprehensive medication review with the FORTA list. However, the study physician is free to decide which medication changes he or she will suggest to his/her patient and the patient is also free to decide which changes he/she wants to be implemented.

To maintain blinding of study participants, it is prohibited for the study physician to tell the study participant about the advisory letter he received from the coordinating center or to show the letter to the study participant. Furthermore, it is important for the blinding of study participants that the study physician in addition provides the patient the information that these medication changes could either mean that he/she is part of the intervention group of the study or that these medication changes were based on the physician’s own judgement because the study physicians are allowed to suggest medication changes as part of usual care also to patients randomized to the control group.

In the control group receiving usual care, medication reviews and changes are not prohibited as they are sometimes done as part of usual care in the German oncologic rehabilitation setting. However, medication reviews are usually not comprehensive by checking the total medication with a medication management software and/or PIM lists. To ensure that intervention and control group are treated differently enough to test the intervention, we will not collaborate with study physicians who conduct comprehensive medication reviews with medication management software or apply a PIM list as part of usual care for older cancer patients.

If no medication changes are discussed in the 2nd study visit, the study physician shall give the study participant the information that this could either mean that the medication review result was that no medication changes are necessary or that they were in the control group. As the exact wording is important in the communication with the patient to avoid accidental unblinding, the coordinating center will provide cards with the text to be communicated from the study physician to the patient in the two different scenarios (Medication changes suggested or not). The study physician shall read the text of the respective card to the patient.

Medication changes to be implemented after the 2nd visit should be documented by the study physician in a standardized form and communicated to the study participants’ general practitioners in the discharge letter. The standardized form should be mailed to the coordinating center.

#### Prescription monitoring

To check adherence of the study physicians regarding the implementation of the medication changes suggested by the FORTA list, a pharmacist in the coordinating center will conduct a second medication review with the FORTA list based on the information from the standardized form documenting the medication changes to be implemented after 2nd visit. Non-adherence of the study physician is defined by a FORTA score at rehabilitation clinic discharge worse by more than 2 points than the FORTA score of optimal medication as assessed by the coordination center in the first medication review. If non-adherence of a single study physician is much higher than that of most other study physicians, he/she will be excluded from further patient recruitment to the study.

It is theoretically possible that learning effects from using the advisory letters about medication changes in the intervention group get translated to the control group (so-called cross-contamination). Thus, all study physicians will be told that it is important that they do not change routine medical care during the study. As a measure to prohibit changes of routine care in the control group, the same prescription monitoring described above for the intervention group will also be applied to the patients of the control group. The average change in FORTA score from the first to the second visit for the first 10 control group participants for each study physician will be used as a baseline. If improvement in the FORTA score increases in control group participants during the study for certain physicians, they will be notified about this and asked to go back to their routine before starting the study. If this is not being done by the study physicians (or if it is not possible for them), they will be excluded from further patient recruitment to the study.

#### Follow-up questionnaires after discharge from rehabilitation clinic (4, 8, and 12 months after baseline)

To obtain primary and secondary outcome information, short follow-up questionnaires will be sent from the coordinating center to the study participants by mail in study months 4, 8, and 12. Two reminders will be mailed to non-responders within 8 weeks after the first sending of a questionnaire at the 4- and 12-month follow-up. For the 8-month follow-up questionnaire, which contains the information of the primary study endpoint, a high completeness rate shall be ensured by additional replacing the second contact reminder via mail by a contact attempts via telephone. The study participants will have the opportunity to give the information asked for in this questionnaire on the phone.

#### Adherence assessment of patients and their general practitioners

The questionnaire at 8-month follow-up will ask the study participant to list all drugs he/she currently uses and if the take ≥ 80% of the prescribed dose. The pharmacist in the coordinating center will perform a third medication review with the FORTA list and calculate the total FORTA score for the medication used with sufficient adherence at the 8-month follow-up. If this FORTA score at the 8-month follow-up is worse than the FORTA score of the medication at the rehabilitation clinic discharge visit by more than 2 points, the patient will be regarded as non-adherent. It should be mentioned that this is not only an adherence assessment for the patients but also for the general practitioners and other caring physicians in the outpatient setting that may prescribe new or different drugs compared to the intended medication after the patient got discharged from the rehabilitation clinic.

#### Survival follow-up (up to 10 years)

The personal data will be regularly compared with data from registration offices and health authorities to monitor the participants’ vital status and to determine the cause of death of the deceased.

### Statistical analysis plan

#### Sample size estimation

A GHS/QoL score difference of 5.86 scale points was shown to be the minimally important improvement [14], and the study is designed with a sufficient statistical power of 80% to detect such a difference or greater in changes of the score between the intervention and control group with p < 0.05. The sample size calculation was conducted with Proc Power, SAS 9.4, SAS Institute Inc., Cary, NC, USA using data from the first n = 38 study participants of the MIRANDA (***Mi****t Dabei!****R****eh****a n****ach****Da****rmkrebs*) study, which is another study, currently being conducted by the coordinating center (https://www.drks.de/drks_web/setLocale_EN.do). The MIRANDA study recruits colorectal cancer patients in German rehabilitation clinics. The change in the EORTC-QLQ-C30 GHS/QoL score from baseline to the 9-month follow-up was approximately normally distributed. For the estimation of mean ± standard deviation (SD) GHS/QoL score change in the control group in the OPTIMAL study from baseline to 8-month follow-up, the mean ± SD change of the GHS/QoL score from baseline to 9-month follow-up in the MIRANDA study was taken. In the MIRANDA study, the mean ± SD GHS/QoL score increased from baseline (52.41 ± 19.17) to 9-month follow-up (60.96 ± 18.89) by 8.55 (± 16.15) score points. For the estimation of mean ± SD GHS/QoL score change in the intervention group, the minimal important difference of 5.86 points was added (8.55 + 5.86 = 14.41) and the SD was assumed to change proportionally ((16.15/8.55)*14.41 = 27.22).

With these assumptions and applying a significance level of 0.05 and 80% power, n = 462 patients need to be randomized 1:1 to the intervention and control group (i.e., n = 231 patients are needed in each group) to detect a score change difference of 5.86 or more points using a two-sided, two-sample, t-test for the mean difference. Taking into account a drop-out rate of 10% (drop-out defined as no follow-up questionnaire available and/or no documented visit with a study physician at the end of rehabilitation), n = 514 patients need to be randomized.

#### Statistical methods

Homogeneity of the intervention and control group will be described by comparison of the demographic data and key baseline characteristics. All statistical tests will have a two-sided significance level of 0.05. In addition, 95% confidence intervals will be estimated for all outcomes in the intervention and control groups. All analyses will be done using SAS software version 9.4 or later.

The primary analysis will test the null hypothesis:

##### H_0_

The change in the EORTC-QLQ-C30 GHS/QoL score from baseline to 8-month follow-up is the same in the two groups.

versus the alternative hypothesis:

##### H_1_

The change in the EORTC-QLQ-C30 GHS/QoL score from baseline to 8-month follow-up is different in the two groups.

The primary endpoint EORTC-QLQ-C30 GHS/QoL score will be analyzed with an intention-to-treat approach, including all randomized patients in the “Full Analysis Set” who had a documented visit at the end of rehabilitation and have any follow-up data (4-, 8- or 12-month follow-up). If data for the primary outcome are missing, multiple imputation of 20 data sets will be conducted using an imputation model including all socio-demographic and health related factors assessed at baseline, 4-, 8- and 12-month follow-up. The Markov Chain Monte Carlo (MCMC) method of the SAS procedure PROC MI will be used for multiple imputation and the SAS procedure PROC MIANALYZE will be used for the analysis, considering the variation between the 20 data sets. In sensitivity analysis, the primary outcome will be imputed for all randomized patients, including subjects with no follow-up data. A per-protocol analysis will be done additionally as a sensitivity analysis excluding all patients with non-adherent study physicians (FORTA score at rehabilitation clinic discharge > 2 points or no 2^nd^ study visit with discussion of medication changes performed before rehabilitation clinic discharge) and non-adherent patients (FORTA score of actually used mediation at 8-month follow-up worse by more than 2 points than the FORTA score of medication at rehabilitation clinic discharge) from the intervention group. In a further sensitivity analysis, subjects who received chemotherapy or radiotherapy or had a tumor surgery in the month prior to the 8-month follow-up questionnaire date will be excluded because this may have a very large impact on their QoL, which could superpose all potential effects of the intervention.

As normal distribution can be assumed for the outcome “change in GHS/QoL score”, the primary test statistic will be the two-sample Satterthwaite’s t-test for the mean difference with unequal variances.

All normally distributed secondary endpoints with a continuous scale will also be analyzed with the two-sample Satterthwaite’s t-test. If normal distribution cannot be assumed, the Wilcoxon rank-sum test will be used. All dichotomous secondary endpoints will be tested with a Chi² test. In addition, a generalized estimating equation model will be applied for all outcomes that will be repeatedly assessed during 12 months of follow-up.

A priori defined subgroup analyses will be conducted for groups defined by rehabilitation clinic, age (65–79 / ≥ 80 years), sex, cancer stage (I or II / III or IV), tumor site (lung, breast, prostate, colorectal, or other), baseline EORTC-QLQ-C30 GHS/QoL score (≤ 52 / > 52 points), baseline drug burden (5–9 / ≥ 10 medications), baseline frailty (robust / pre-frail / frail), and use of anti-depressant or anti-psychotic medication at baseline (yes/no).

Because of the multiplicity of tests for subgroup and secondary outcome analyses, all results except for the primary endpoint, will be regarded as exploratory and not confirmatory.

No interim analyses are planned.

### Data management & protection

The names of the patients and all other confidential information are subject to medical confidentiality, the General Data Protection Regulation (Datenschutz-Grundverordnung (DSGVO)) and the Federal / State Data Protection Law (Bundes-/Landesdatenschutzgesetzes (BDSG / LDSG BW)). The appropriate regulations of local data legislation will be fulfilled in its entirety. All data obtained in the course of the study will be treated pursuant to the Federal Data Protection Law (BDSG) and the European ordinance (EU) 2016/679 (Datenschutz-Grundverordnung).

Patient data will be documented anonymized/pseudonymized and may only be passed on to universities, clinics, or companies in anonymized/pseudonymized form. Third parties do not have access to original documents. A decoding of the pseudonymization will only be conducted in case of complete data deletion for study participants who demanded it.

Study data stored on a computer will be stored in accordance with the local data protection law and will be handled in strictest confidence. Distribution of these data to unauthorized persons will be strictly prevented. The only persons who have access to the pseudonymization code are the study’s medical documentarist and his/her deputy who is also a medical documentarist and is not involved data analyses.

A data monitoring committee, monitoring of study centers and a trial auditing system will not be implemented because the trial will not be conducted according to the laws of a drug trial. This is also the reason, why no data on adverse events or other unintended drug effects will be collected and no provisions for post-trial care are made.

### Patient questionnaires, declaration of consent, and transfer agreement

The questionnaires to be completed by the study participants are marked with a unique identification number (= study participant’s ID). Study participants are asked to give their name, address, date of birth, phone and mobile number on the questionnaire cover sheet. After receipt of the questionnaire at the coordinating center, the cover sheet with the personal data is separated from the rest of the questionnaire, which only contains the ID.

The further storage and processing of the personal data will be carried out completely separately from the processing of the study data. The cover sheet of the questionnaire is stored exclusively in a separate steel cabinet in a room with a special locking system. Only a limited group of appointed persons has a key to this room. The personal data on the cover sheet are processed on a computer located in the coordinating center. This computer contains the software as well as the files for administering the study participants. Access to this computer is restricted to the persons responsible for organizing the study. The computer is secured by a special security software, for which a special password is required in order to gain access.

The declaration of consent contains the name and signature of the participant. After receipt at the coordinating center, it will be kept in the same way as described for the cover sheet of the questionnaire (see above).

### Blood samples

The samples will be collected, transported, stored and processed/analyzed in pseudonymized form, provided only with the unique study participant’s ID.

### Long-term data storage & anonymization

All study data and samples will be stored in a biobank at the German Cancer Research Center for a minimum of 20 years after the end of recruitment. Subsequently, it will be determined whether further storage is necessary. If no scientific data analyses were conducted over a 5-year period after the minimum storage time, the study data will be deleted. Person identifying data will be stored for a maximum of 15 years to complete the 10-year survival follow-up (10 years of follow-up time + 3 years of recruitment time + 2 years of data availability delay). As soon as vital status and cause of death needed for 10-year survival were ascertained, the personal data (names, addresses, birth date and phone numbers) will be deleted. A subsequent assignment of samples and data to a specific person is then no longer possible (= anonymization).

## Discussion

We will assess whether an advisory letter with the results of a comprehensive medication review conducted with the FORTA list to the treating physician in rehabilitation clinics improves the global QoL of older cancer patients exposed to polypharmacy more than usual care in a multi-center, single-blinded RCT. QoL is a highly patient-relevant outcome for older cancer patients [15, 16]. Furthermore, multiple secondary endpoints, specifically relevant for the elderly, will be tested, such as physical functioning, cognitive functioning, fatigue, frailty, falls, hospitalizations, and survival. The RCT is novel in multiple aspects:

- First RCT with a QoL score as the primary endpoint and not as a secondary endpoint.
- First RCT in the oncological rehabilitation setting.
- First trial applying the FORTA list in older cancer patients.

In recent years, increasing efforts have been made to reduce PIP by implementing medication reviews in older cancer patients. Recent studies presented beneficial effects of medication reviews with respect to reducing PIP rates and functional assessment scores in cancer patients [17–23]. However, evidence for the effects on patient-relevant endpoints, such as QoL, hospital (re-)admissions, or mortality is sparse because previous studies with older cancer patients were mostly feasibility-oriented (to test if implementing medication reconciliation in oncology ambulatory settings is feasible), underpowered, with relatively short follow-up periods, or they did not record the mentioned patient-relevant outcomes.

Effectiveness concerning patient-relevant endpoints is largely unknown and neither proven in RCTs for underuse [24], overuse, nor PIM use [25–28]). The FORTA list is no exception because the primary outcome tested in the VALFORTA-RCT with 409 hospitalized older adults was medication appropriateness and this outcome was significantly improved [29]. However, application of the FORTA list also improved patient-relevant secondary outcomes in that trial, which were the total number of adverse drug reactions, limitations in activities of daily living and renal failure. This distinguishes the available evidence for the FORTA list from that of most other drug lists. However, it needs to be mentioned that the VALFORTA trial had methodological weaknesses, including problems with the randomization leading to unbalanced patient characteristics among the two groups, no correction for multiple testing when analyzing multiple secondary outcomes and a lack of a per-protocol-analysis. Thus, it is important to check the results of the VALFORTA trial in another trial applying the highest methodology standards of RCTs.

However, despite its methodological weaknesses, the VALFORTA trial’s results are encouraging to use the FORTA list in further RCTs. Other reasons for choosing the FORTA list include aspects of feasibility to translate the study’s results into regular care. FORTA is being updated annually. Although it was specifically designed for the German drug market it is increasingly being made available for other countries like the US and Japan [30, 31]. Moreover, there is a smartphone app specifically designed for the FORTA list, which allows the healthcare providers to apply it more efficiently. The app is free of charge and is quickly used. It takes approx. 5–10 min to put in information about a patient and to obtain the recommendations for medication changes.

Thus, if the RCT is successful and shows that the comprehensive medication review with the FORTA list has an impact on the patients’ QoL, the trial results could easily be translated into the routine of care for older cancer patients. As the FORTA list and smartphone app are free of charge, only the time the physician spends to do the medication review is a cost issue. We are very confident that the medication review with the FORTA list is a highly cost-effective measure in the care for older cancer patients exposed to polypharmacy because it should be able to improve drug safety by preventing adverse drug events (by reduction of PIP, drug-drug interactions, and drug overdosing), by maximizing therapeutic effectiveness (by avoiding medication underuse), and by reducing unnecessary costs for drugs (by omitting duplicate prescriptions or prescriptions of drugs without proven efficacy in older adults).

We think, that the results of the OPTIMAL trial will be generalizable to other developed countries with comparable cancer care as in Germany. Although other health care systems may not have rehabilitation clinics for oncological care, they will have other institutions caring for older cancer patients after primary cancer therapy, like oncological specialists’ practices, in which the medication review with the FORTA list could be implemented.

Results of the OPTIMAL trial will be presented in international meetings and published following the CONSORT statement in a high-ranked international medical peer-reviewed journal, independent of the results. Moreover, all oncological rehabilitation clinics in Germany will be informed about the results per letter to ensure effective translation into practice. Lastly, patient advocacy groups for cancer patients in Germany will be informed about the trial’s results for further distribution among their members.

## Data Availability

The data will not be made available in an Open Access Platform. After completion of the study, interested scientists can request data and can receive pseudonymized data upon approval of this application by the principal investigator of the OPTIMAL study (Dr. Ben Schöttker; b.schoettker@dkfz.de) and a signed data transfer agreement with the DKFZ.

## List of abbreviations

EORTC QLQ-C30: European Organisation for Research and Treatment of Cancer’s Quality of Life Questionnaire’s Core 30 items, EORTC QLQ-FA12
FORTA: Fit fOR The Aged
GHS: Global Health Status
ICD: International Classification of Diseases
MIRANDA: Mit Dabei! Reha nach Darmkrebs
OPTIMAL: Optimization of polypharmacy in geriatric oncology
PIM: potentially inappropriate medication
PIP: potentially inappropriate prescribing
QoL: Quality of life
RCT: randomized controlled trial
SD: standard deviation
SPIRIT: Standard Protocol
Items: Recommendations for Interventional Trials
